# 非小细胞肺癌靶向治疗的靶点研究进展

**DOI:** 10.3779/j.issn.1009-3419.2013.02.09

**Published:** 2013-02-20

**Authors:** 洪省 薛, 少华 周, 万鹏 卢, 志龙 赵

**Affiliations:** 116001 大连，大连大学附属中山医院心胸外科 Department of Thoracic Surgery, Zhongshan Hospital, Dalian University, Dalian 116001, China

目前，肺癌是恶性肿瘤死亡的首要原因，约占全世界所有癌症死亡人数的1/3。非小细胞肺癌（non-small cell lung cancer, NSCLC）在肺癌中约占80%-90%^[1]^，严重威胁人类健康，其中30%-40%的患者在确诊时已属晚期而失去手术机会。晚期NSCLC若不接受治疗，中位生存期为4个月-5个月，1年生存率小于10%^[2]^。以铂类药物为基础的系统化疗在一定程度上延长了患者生存期（中位生存期为8个月-9个月，1年生存率为30%-40%），改善了患者症状，但有效率小于30%。近年来，随着医学分子生物学的发展，分子靶向治疗日益受到临床重视，已经成为恶性肿瘤治疗的新手段^[3]^。针对NSCLC的靶向治疗研究不断深入，有关基础及临床研究不断涌现。本文旨在对NSCLC相关靶点的基础研究成果进行总结。

## 1 表皮生长因子受体（epidermal growth factor receptor, EGFR）

EGFR已经成为比较明确的药物靶向治疗目标^[4]^。EGFR为广泛分布于人体各组织细胞膜上的多功能糖蛋白，是酪氨酸激酶（TK）信号通路中的重要分子。EGFR位于第7号染色体p13-p22区，由28个外显子组成，编码1, 186个氨基酸，其分子蛋白量约170 kDa。EGFR由胞外配体集合区、跨膜输水区、胞内酪氨酸激酶区等3部分组成。胞外区的配体是可溶性或膜结合的多肽或蛋白类激素，胞内区为ATP结合位点和保守的酪氨酸激酶区。活化的EGFR通路主要通过有丝分裂原活化蛋白激酶、磷脂酰肌醇3位羟基激酶、信号转导物和转录激活子蛋白3及5等通路引起一系列细胞反应，包括细胞的增殖、分化、信号的传导及血管的形成、抑制细胞凋亡等^[5, 6]^。

EGFR在45%-85%NSCLC中阳性表达^[7, 8]^，且在正常组织、癌旁组织、及癌组织中的表达呈递增趋势。通过DNA测序法、聚合酶链式反应-酶切法、TaqMan-MGB探针实时聚光聚合酶链式反应法、高效液相色谱法等检测组织中*EGFR*的突变均可得到满意结果。

*EGFR*突变的方式主要有EGFRv Ⅰ、EGFRv Ⅱ、EGFRv Ⅲ。突变的*EGFR*在没有配体存在的情况下，有不依赖配体的、持续的酪氨酸酶活性。Lynch等和Paez等率先报道了NSCLC中*EGFR*突变是靶向药物治疗奏效的一个必要前提。肿瘤对靶向治疗药物敏感则主要在于*EGFR*的突变，在所有患者中约有24.5%发生*EGFR*突变^[9]^。使用特异性的酪氨酸激酶抑制剂（TKI）阻断EGFR细胞内传导通路后将会阻断肿瘤细胞的生长、转移或诱导凋亡，且能明显提高生存率^[10]^。*EGFR*突变主要发生于19区的746-752位密码子缺失和21区的第858位密码子点突变。约有20%患者对EGFR-TKIs不敏感或者无效，其机制主要包括外显子20插入突变及T790M突变，*KRAS*激活突变，*PTEN*缺失等最终产生耐药性^[11]^，而且EGFR并不依赖于高浓度的EGFR-TKIs^[12]^。研究^[13]^发现双重抑制肝细胞生长因子和血管内皮生长因子可以获得解除耐药性的问题。EGFR的表达程度及胞内信号传导通路的活性程度与EGFR-TKIs治疗有密切关系^[14]^。寻求关于EGFR耐药性的基础研究将对NSCLC治疗更为重要。

## 2 血管内皮生长因子（vascular endothelial growth factor, VEGF）

在发生血液转移的肿瘤中VEGF含量明显增高^[15]^。肿瘤的转移和生长依赖于血管的形成，VEGF是内皮细胞有丝分裂过程中高度特异的生长因子，因其在肿瘤血管形成过程中起重要作用，成为人们关注的焦点^[16]^。编码VEGF的单个基因能编码121、145、165、183、189和206等6个亚型的氨基酸^[17]^，研究还发现VEGF 165含量和活性较其余亚型高。

VEGF是通过与VEGFR结合而发挥作用的，VEGFR作为酪氨酸激酶家族的一个成员，包括：VEGFR1（FIT-1）、VEGFR2（KDR/FLK-1）、VEGFR3（FIT-4）、Neuropilin-1（NRP-1）。它们均含有7个免疫球蛋白样结构的细胞外区、膜区和酪氨酸激酶区，均为跨膜受体。受体催化域内有酪氨酸激酶插入区，通过受配体结合引起细胞内酶联反应。VEGF-A可以与VEGFR1、VEGFR2、Neuropilin-1受体结合，促进血管血管内皮有丝分裂，介导血管生成。VEGF-B选择性与VEGFR1结合，调节血管外基质降解、细胞迁徙及粘附作用，介导血管生成。VEGF-C、VEGF-D与VEGFR2、VEGFR3结合诱导淋巴管内皮细胞有丝分裂、增殖，介导淋巴管生成^[18]^。通过配体和受体的结合，发挥其促肿瘤作用^[19]^。

通过肿瘤组织、血液学以及支气管灌洗等获取患者VEGF，并进行定量检查，能够指导临床用药及预后判断^[20]^。研究^[21]^表明在NSCLC中高水平的VEGF表达提示NSCLC预后差。肿瘤细胞通过在缺氧敏感细胞中激活低氧诱导信号通路触发VEGF表达，VEGF不仅在肿瘤细胞表达，肿瘤相关的基质细胞中也有表达，通过分泌大量VEGF刺激新生血管形成以满足生长所需能量的需求^[22]^。VEGF、VEGFR在NSCLC中表达率分别为54%和60%^[23]^，提示VEGF的过表达促进了NSCLC的生长和增殖。肿瘤的生长必须依赖足够的血供，切断肿瘤血供则可抑制肿瘤生长，导致肿瘤死亡，因此抗肿瘤血管生成成为治疗肿瘤的一个重要靶点。已有大量研究^[24]^通过抑制VEGF释放、使用单克隆抗体结合释放的VEGF、封闭VEGFR等方法阻断VEGF与VEGFR结合，阻断新生血管和淋巴管的形成，从而抑制肿瘤细胞生长。目前针对VEGFR的多重酪氨酸激酶抑制剂已经用于治疗晚期肺癌细胞肺癌并取得了满意的临床效果^[25]^。

通过阻断VEGF信号通路已经初步达到了抗肿瘤的效果，然而部分患者对VEGF抑制剂产生耐药性^[26]^，而且在小鼠的肺癌动物模型中出现了计量和时间相关性。在副反应可以耐受的情况下足够剂量的VEGF抑制剂可以达到满意的效果^[27]^。更重要的是VEGF抑制剂不仅抑制了肿瘤血管生成，正常血管生成也受到损害，而且还有出血和血栓形成的危险^[24]^，这也使得VEGF抑制剂的发展受到了一定的阻滞。研究者正努力寻求新的抗肿瘤血管形成的特殊靶点，希望能够不影响正常血管的形成而又达到抗肿瘤的目的。

## 3 棘皮动物微管相关蛋白4-间变性淋巴瘤激酶（echinoderm microtubule-associated protein 4-anaplastic lymphoma kinase, EML4-ALK）

*EML4*-*ALK*是肺癌诱发基因之一，属于融合基因，该基因在NSCLC中的表达率约为3%-8%^[28]^，EML4-ALK现已成为新的NSCLC治疗靶点^[29]^。大量Ⅰ期和Ⅱ期试验数据证实ALK抑制剂具有稳定的抑制肿瘤的作用^[30]^。*EML4*-*ALK*融合基因N端为EML4编码，C端为ALK编码，在2号染色体的短臂上转化而成^[31]^。目前已证实EML4包含卷曲螺旋区域的部分与ALK形成二聚体后间接激活该融合基因^[32]^，从而激活下游异常信号通路，导致肿瘤发生在间变性大细胞瘤中，EML4-ALK已被证实能调节下游RAS-ERK、JAK3-STAT3、PI3-K/AKT等信号通路，导致细胞增殖、永生化和骨架及外形的改变^[33]^。在NSCLC中鉴定出10余种*EML4*-*ALK*融合基因变异体，而且还有融合了TFG和KIF5B的变异体，且所有变异体均有生物学功能^[34]^。ALK阳性的肿瘤多为腺癌特别是印戒细胞癌^[35, 36]^。Soda等^[37]^研究了75例NSCLC患者，首次检测到其中5例表达EML4-ALK嵌合蛋白，Rikova等证实了该项研究。

*EML4*-*ALK*融合基因目前主要通过聚合酶链式反应、荧光原位杂交、免疫组织化学等方法检测。通过实验发现ALK抑制剂具有积极的抗肿瘤作用，可能是由于*ALK*基因5′末端缺失引起的^[38]^。再有研究^[39]^发现EML4-ALK与*EGFR*、*KRAS*突变不同时存在，但在NSCLC中常存在一些共同临床特征：年轻的不吸烟或少吸烟者、病理为腺癌者等。但是这些特征并不是存在于所有的国家，调查发现在老年吸烟的人群中也有发生ALK融合。所以临床特点不足以检查*ALK*突变状态，进行相关的分子学检测是必要的^[40]^。

EML4-ALK作为新的肿瘤治疗靶点——通过小分子抑制剂抑制ALK酪氨酸激酶区域的活性，阻断其下游信号传导通路，从而抑制了肿瘤细胞的增殖和生长，达到控制肿瘤的目的。大量临床研究^[41]^证明ALK抑制剂治疗ALK阳性的肺癌患者取得了明显的效果。与此同时NSCLC中ALK阳性的患者使用ALK抑制剂并不能阻止疾病的进展，有以下几种耐药机制解释：①ALK激酶突变；②*ALK*基因重组拷贝数增多；③*EGFR*/*KRAS*突变。还有一部分则是对治疗起初就无任何反应^[42]^。对于ALK抑制剂的治疗适应症尚缺少准确的标准^[39]^，但是检测肿瘤患者的ALK阳性率对于判断其融合阈值非常重要。对于EML4-ALK的研究已经有了新的认识，更深的认识有待进一步探索。

## 4 胰岛素样生长因子受体（insulin-like growth factors receptor, IGF-1R）

IGF-1R是一种四聚体糖跨膜糖蛋白，具有酪氨酸激酶活性，与配体IGF结合后，激活下游信号通路，介导肿瘤细胞中多条信号传导通路，直接影响肿瘤细胞的有丝分裂、分化和迁徙，进而促进肿瘤细胞的生长和转移^[43]^。研究^[44]^发现IGF-1R的糖基化程度直接影响其介导的肿瘤分化及转移过程。高水平的IGF-1R表达在癌细胞中表现为恶性度高和预后差^[45, 46]^。IGF-1R在NSCLC中的表达率为30.5%^[47]^。有研究^[48]^证实封闭IGF-1R能致活体内肿瘤细胞大量凋亡，抑制肿瘤发生，阻断远处转移。

IGF-1R是由2个α亚基和2个β亚基构成的寡聚蛋白，亚基与亚基之间通过二硫键连接，可以与IGF-1和IGF-2结合，且与胰岛素之间有交叉反应，但亲和力比前二者低100倍-1, 000倍。在细胞内IGF-1R与配体结合后激活IGF-1R信号通路，激活的IGF-1R信号通路可以激活酪氨酸激酶，然后再通过激活磷脂酰肌醇3激酶/丝氨酸苏氨酸蛋白激酶通路和丝裂原活化蛋白激酶（mitogen-activated protein kinase, MAPK）通路而发挥抗凋亡、促进细胞增殖分化等作用^[49]^。此外IGF-1R的信号转导还可以促进细胞向恶性转化并改变细胞的粘附性^[50]^。

癌组织中IGF-1的浓度较癌旁组织中明显增高^[51]^，虽然肺癌细胞中*IGF*-*1*基因表达频率很高，但是只有NSC-H417D细胞株才有IGF-1的肽段。高浓度的IGF-1可以抑制NSCLC细胞株A549的生长，可能是由于IGF-1的浓度和肺癌暴露在IGF-1的时间不同，使PI3K信号通路持续性激活，以及诱导P21增加所致。所有NSCLC细胞均可表达IGF-1R。Trop-2作为细胞表面糖蛋白表达明显降低，对于IGF-1R和IGFs的结合的抑制降低，表达IGF-1R的癌细胞通过合成和分泌IGFs，受配体结合后刺激癌细胞无限增殖，维持其恶性表型^[52]^。

研究^[53]^表明，通过抗IGF-1R抗体、IGF-1类似物以及反义RNA使IGF-1R功能失活或数目减少，均可以导致相应的癌细胞系大批凋亡，阻止体外细胞增殖。Zia^[54]^用体外试验方法用100 ng/mL的IGF-1可使NSCLC细胞株NCI-H1299的生长增加7倍。另有报道^[55]^通过应用特异性联合抑制IGF-1R/IGF-2 mRNA反义脱氧核苷酸治疗NSCLC，对癌细胞的生长抑制作用大于80%，说明IGF-1R的反义治疗可以成为一种有效的靶向治疗方法。目前对于抗IGF-1R信号通路治疗仍处于初级阶段，对于IGF-1R抗体和小分子的酪氨酸抑制剂二者对于治疗NSCLC的疗效并不确切，更进一步的尝试有待努力。

## 5 组蛋白去乙酰化酶（histone deacetylases, HDACs）

HDACs作为具有良好前景的抗肿瘤靶点日益受到研究者的关注^[56]^，HDACs主要通过诱导染色体重塑，抑制基因转录，从而达到抗肿瘤的目的^[57]^。HDACs参与DNA复制和DNA损伤应答，作用于细胞周期的相关因子，影响细胞增殖与分化，作用于细胞凋亡相关蛋白，影响凋亡过程，联合血管生长因子，促进肿瘤组织血管移行与形成等^[58]^。在NSCLC组织中HDACs表达量较癌旁组织明显增高^[59]^。HDACs表达与肺癌进展和对药物耐受相关，并且作为NSCLC不良预后的独立影响因子^[60]^。通过小分子干扰RNA或者特殊抑制剂作用HDACs阳性肿瘤细胞可以使细胞停留在G_1_期或者G_2_/M转变期，结果造成细胞不能正常进行有丝分裂，细胞生长受到抑制，凋亡增加^[61]^。

组蛋白参与构成染色质，其中核心组蛋白是由一个球形结构域和一个延展性且进化上保守的N端尾部，这些N端尾部是调节生命活动的重要信号通路作用靶点。正常生理状态下组蛋白乙酰基转移酶（histone acetylase, HAT）与HDACs对组蛋白乙酰化处于平衡状态。细胞在发生转化的状态下HDACs活性明显增强，使原有平衡被打破，导致一些影响细胞增殖和调控细胞周期的分子表达失衡，影响转录因子、信号转录介质、DNA损伤修复酶、伴侣蛋白和结构蛋白等功能^[62]^。除了调节组蛋白的转录状态，HDACs还作用于一些与肿瘤发生相关的非组蛋白，例如p53、E2F1、а-tubulin、HIF-1α和HSP90。HDACs由18个基因组成，主要分为4大类，其中Ⅰ类、Ⅱ类和Ⅳ类属于Zn^2+^依赖的HDACs；Ⅲ类属于DNA^+^依赖的HDACs^[63]^。其中Ⅰ类包括HDAC1、2、3和8几个亚型主要定位在细胞核。Ⅱ类与酵母蛋白基因*hda1*同源，包括HDAC4、HDAC5、HDAC6、HDAC7、HDAC8、HDAC9和HDAC10几个亚型，穿梭于细胞核和胞浆之间。Ⅲ类与酿酒酵母菌沉默蛋白基因*Sir2*同源，广泛分布于细胞中。Ⅳ类主要是HDAC11定位于细胞核^[64]^。

HDACs在不同的肿瘤中表达存在差异，其大多数亚型在多种肿瘤组织中高表达。其中HDAC1在肺癌中高度表达^[65]^。研究^[66]^发现HDAC5在NSCLC组织较相应的癌旁组织表达明显降低，提示HDAC5在NSCLC的发生发展中起重要作用，可以通过免疫组化的方法检测NSCLC组织和癌旁组织中HDACs的表达。随着对HDACs在NSCLC治疗中的重视，有关HDACs的研究将进一步深入。

## 6 Raf激酶及其介导的Raf/MEK/ERK信号通路

目前已知所有真核细胞中均存在Raf/MEK/ERK通路，其通过Ras、Raf、MEK、ERK的磷酸化将级联放大的信号由胞外传入细胞核内^[67]^。Raf可以通过或者不通过Ras的方式发挥其信号转导调节作用。作为Raf激酶的下游底物激活的MEK磷酸化ERK作用多种底物以调节细胞功能^[68]^。细胞的增殖、生长、分化、迁徙被不同的细胞核内的信号通路所控制，在这些所有的信号通路中Raf激酶及其介导的Raf/MEK/ERK信号通路在介导人类NSCLC中的作用已经被确认^[69]^。研究^[70, 71]^发现使用Raf/MEK/ERK信号阻断剂能够有效的阻断肿瘤细胞中Raf/MEK/ERK信号通路，抑制肿瘤细胞的生物学功能。

Raf激酶的3个同工酶包括A-Raf、B-Raf、C-Raf，与细胞增殖、分化、生存、附着以及血管生成的调节密切相关。C-Raf在大多数人体组织中表达，且具有不通过Raf/ERK/MEK通路即可调节细胞的功能，在NSCLC中异常激活^[72]^。B-Raf、C-Raf除参与肿瘤形成外，与新生血管的形成密切相关。C-Raf与NSCLC发生有密切关系：首先，Ras作为C-Raf上游基因在部分NSCLC中存在突变；其次，NSCLC中*C*-*Raf*基因过度表达，C-Raf激活上调；此外，C-Raf下游基因MEK的过度表达可抑制细胞凋亡，从而导致肺癌细胞过度表达^[73]^。对Raf激酶进行抑制可成为NSCLC靶向治疗的有效途径^[74]^。

Raf激酶抑制蛋白（PKIP）属于高度保守的磷脂酰乙醇胺结合蛋白家族，广泛存在于多种生物中。PKIP参与Raf-MEK1/2-ERK1/2、G蛋白、NF-қB等多条信号传导通路的调节，还通过G蛋白偶联受体激酶-2来发挥受体信号调节器的作用^[69]^。研究^[75]^发现PKIP具有抑制NSCLC转移的作用，可能成为治疗NSCLC侵袭转移的新靶点。随着Raf/MEK/ERK信号通路研究的进一步深入，该信号通路将成为潜在的NSCLC治疗靶点。

## 7 蛋白激酶Ⅱ（casein kinase 2, CK2）

CK2是一种在真核细胞中普遍存在的高度保守信使非依赖性丝/苏氨酸蛋白激酶^[76]^。CK2是一种多功能蛋白，对细胞生长、增殖、凋亡等方面均具有很重要的调控作用^[77]^，其表达水平精密地调控着正常细胞的生物学活性，其表达失衡与多种肿瘤发生相关，其中包括NSCLC。高水平的CK2标志着多种肿瘤预后不良，而且CK2影响多种细胞内信号通路，比如PI3K、NFkB和Wnt等^[78]^。通过CK2抑制剂抑制CK2的表达能够明显降低肿瘤细胞的生长和代谢^[79]^。

CK2是一类酶家族，由两类催化亚基和一类调节亚基构成。CK2的调节机制不像其它激酶受严格的开/关激酶调控，而是存在更精确的调控机制，如磷酸化、蛋白之间的相互作用等，这些调节使CK2的调控更加准确^[80]^。迄今为止，在已检测过的肿瘤中CK2表达均失调。研究^[80]^发现过表达CK2а联合c-myc的转基因表达可以明显增加小鼠淋巴瘤和白血病的发病率，抑制CK2а表达可明显降低疾病发生。CK2可以影响c-myc的稳定性，并且能作为肿瘤发生相关的Wnt信号的正性调节物^[81]^。CK2与细胞凋亡的关系密切，是细胞凋亡的重要控制点。CK2通过多种细胞核和胞质中的靶点参与多条途径，发挥各种整体的、精细的调控作用，从而抑制细胞的凋亡，维持肿瘤细胞生长。

NSCLC中CK2表达明显增高，mRNA表达水平与激酶活性基本平行。癌旁正常组织较癌组织中CK2а表达高，同腺癌相比，鳞癌中CK2β表达增高。研究^[82]^证明CKа是肺鳞状细胞癌的独立预后影响因子，提示CK2可能是NSCLC的特殊生物学标志物。通过抑制CK2活性为NSCLC靶向治疗提供新的途径。研究^[83, 84]^发现早幼粒细胞白血病蛋白（promyelocytic leukemia, PML）具有控制细胞生长、诱导凋亡、促进细胞老化，在NSCLC中PML发生缺失造成肿瘤发生。研究^[85]^发现在NSCLC中CK2а与PML的表达呈反比。CK2通过调控PML，引起其转录后缺失，从而导致其肿瘤抑制功能丧失^[86]^。目前关于CK2的基础研究正逐步深入，CK2将成为治疗肿瘤潜在的多目标靶点。

## 8 总结

肺癌的靶向治疗近几年取得了一定的进展，但是大多数肺癌患者的生存时间仍未明显提高。对于肺癌分子基础的不断研究已经使人们对肺癌发生、发展过程中失调的信号通路网络有了更深入的认识。其中表皮生长因子受体激酶抑制剂gefitinib、血管内皮生长因子抑制剂bevacizumab、棘皮动物微管相关蛋白4-间变性淋巴瘤酶抑制剂crizotinib、胰岛素样生长因子受体抑制剂、干扰组蛋白去乙酰化酶表达、抑制Raf激酶活性阻断其介导的Raf/ERK/MEK信号通路、干扰蛋白激酶CK2表达等研究及药物开发在基础实验和临床应用中均取得了很大的进步。然而，还存在其它未知的或者了解得并不透彻的机制。只有深入充分地了解肺癌的发生、发展及其抗药机制，才能根据不同的肺癌分子改变开发出具有针对性的治疗药物，并有效地应用于特定的患者群体，发展并完善肺癌的个性化治疗。同时，还有众多相关靶点研究未纳入本文，如*SOX2*基因、*P63*基因、KEAP1和NFE2L2通路、纤维母细胞生长因子受体（fibroblast growth factor receptor, FGFR）、盘状结构域受体2（discoidin domain receptor2, DDR2）等与NSCLC密切相关。在多学科综合的不懈努力下将使人类不断地向开发更加有效和毒性更小的抗NSCLC治疗目标迈进。
